# Household quarantine of second degree contacts is an effective non-pharmaceutical intervention to prevent tertiary cases in the current SARS-CoV pandemic

**DOI:** 10.1186/s12879-021-06818-w

**Published:** 2021-12-19

**Authors:** Josef A. I. Weigl, Anna-Katharina Feddersen, Mona Stern

**Affiliations:** Public Health Institute Ploen, Schleswig-Holstein, Hamburgerstr.17/18, 24306 Ploen, Germany

**Keywords:** Asymptomatic, Contact tracing, Isolation, Lockdown, SARS-CoV2, Transmission

## Abstract

**Background:**

Given the characteristics of SARS-CoV2 with regard to transmission before the onset of symptoms and varying manifestation indices according to age, isolation and quarantine have limited efficacy in the current pandemic. Household quarantine in second degree contacts (Hh-Q2°) outside the case household has so far only been addressed by modellers. In the literature there is no publication based on field data.

**Methods:**

In a retrospective cohort study on real field data from a county health department (CHD), all PCR-confirmed cases and related contact persons put into quarantine were analysed. Hh-Q2° was used in our CHD from the onset of the pandemic.

**Results:**

From 9 March to 8 December 2020, 353 PCR-confirmed cases were registered in the CHD Ploen, Northern Germany: 225 (63.7%) primary, 107 (30.3%) secondary and 21 (5.9%) tertiary cases. The 107 secondary cases resulted out of 470 (22.8%) close or 1°contacts and 21 tertiary cases out of 179 (11.7%) indirect or 2°contacts put into quarantine. The efficacy of Hh-Q2° was 51.5% (11.7%/22.8%) of that of quarantine in 1°contacts; 16.4% of all converted cases in quarantined persons were ascertained by Hh-Q2°. One in ten 1°contacts in households with tertiary cases remained asymptomatic.

**Conclusion:**

The impact of Hh-Q2° in preventing further spread of SARS-CoV2 was considerable. With half the conversion rate in 2°contacts compared to 1°contacts, the efficacy of Hh-Q2° is substantial. Hh-Q2° should definitely be used routinely to control the spread of SARS-CoV2 more efficiently and national authorities should include it in their guidelines.

## Background

Until the recent launch of immunization against SARS-Coronavirus-2 (SARS-CoV2), control measures for the current pandemic have relied on non-pharmaceutical interventions (NPI). Isolation of cases and quarantine of contact persons are measures focusing on individuals or households. Given the parameters of SARS-CoV2, such as fraction of asymptomatic cases and transmission before the onset of symptoms, isolation and quarantine are of limited efficacy and can quickly become even more inefficient if not carried out as fast and as comprehensively as possible [1–4]. Therefore, utmost effort is needed to increase the efficacy of isolation and quarantine. The former depends on the public health strategy and available resources for testing, the latter on the power of the local health departments. Household quarantine (Hh-Q) in general and Hh-Q of second degree contacts (Hh-Q2°) as a NPI tool in particular is not consistently used in the current pandemic. So far, the Robert Koch-Institute (RKI) as national centre for disease control in Germany, recommends putting first degree contacts or close direct contacts (1°contacts) into quarantine. Exposure of the other household members by 1°contacts, however, continues across the entire period of the quarantine of the 1°contacts at home, once the 1°contact starts shedding and transmitting, whether becoming symptomatic or not. Thus, the risk of not breaking the chain of transmission by only putting 1°contacts into quarantine instead of the entire household is obvious. In our county health department (CHD), Hh-Q2° has been used since the onset of this pandemic.

The aim of this paper is to investigate and demonstrate the impact and efficacy of Hh-Q2° to prevent tertiary cases and finally raise the efficacy of NPI.

## Methods

**Fig. 1:**
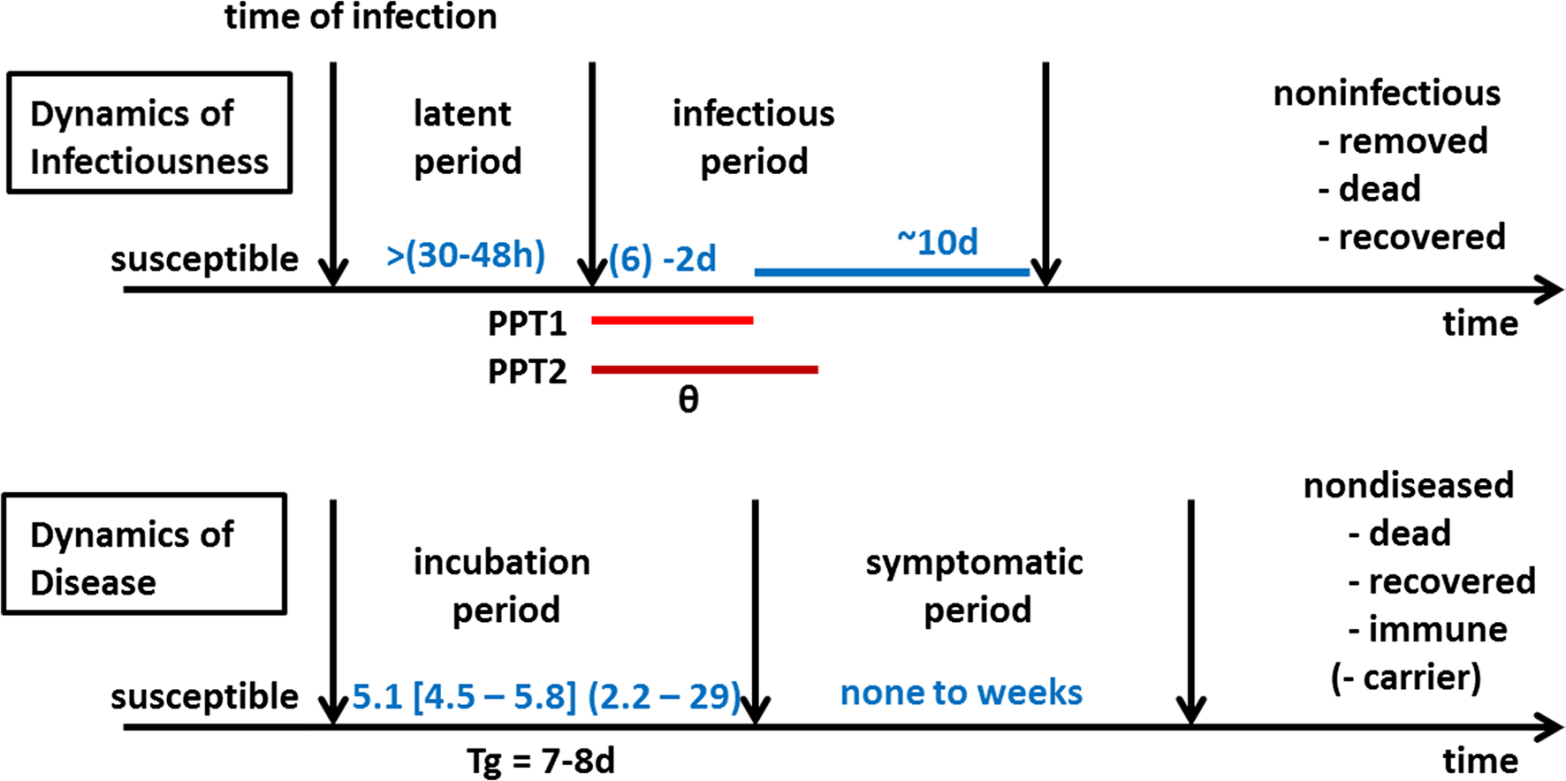
Critical timelines of SARS CoV2 from the perspective of the virus and the host, respectively (in analogy to Halloran [6]). The parameters for SARS-CoV2 are inserted into the didactic figure by Halloran within the referring chapter in the classical textbook by Rothman and Greenland [6]. Dynamics of infectiousness refers to the perspective from the virus. Dynamics of disease refers to the host, the human. Tg generation time; PPP1 prepatency period 1: time from start of viral shedding to start of symptoms [5]; PPP2 prepatency period 2: from start of viral shedding to isolation [5]; θ: fraction infected before onset of symptoms or isolation, respectively [1]; incubation period according to Lauer et al. [7]

In a retrospective cohort study, all confirmed cases under the responsibility of the County Health Department Ploen (CHD Ploen) and related quarantine orders triggered by the primary cases notified between 9 March and 8 December 2020 according to the German Infectious Diseases Control Act (IfSG), were eligible and analysed. In the CHD Ploen, each PCR-confirmed case receives a consecutive case number. Cases #1 to #353 were investigated based on the referring in-house database and the source documents at the time of contact tracing including information on household members. The duration of quarantine during this time period was 14 days according to the guidelines of the Robert Koch-Institute (RKI) (www.rki.de). A quarantine of entire households can be ordered, if a case, finally confirmed by PCR, is diagnosed in a given household. These household members by definition would be 1°contacts, i.e., close direct contacts. By definition of the RKI, a close contact would be for more than 15 min duration and less than 1.5 m distance without personnel protective equipment (PPE). Since a discussion concerning aerosols came up later and was only integrated into the contact definition as of October 19, 2020, it is omitted here. The intensity of a 1°contact can be close (contact person type 1) or not as close (contact type 2) or under PPE (contact type 3). Often the primary case, most often also the index case, has 1°contacts outside of his own household (e.g., at work). The household members besides the 1°contact(s) of a non-case household would be so-called 2°contacts, who by definition only had indirect contact to the primary case via the household member who was a 1°contact. In case the household members cannot separate themselves from the close contact within 72 h (60–96 h) of first exposure to the index case or separate at the time the close contact is tested negative, the entire household can incubate the virus so that tertiary cases might occur beyond the secondary cases in 1°contacts. Transmission from the 1°contact can well start within the so-called prepatency period, the time window of viral shedding before onset of symptoms (i.e., before the end of the incubation period) [5]. Beyond this, the 1°contact may or may not become symptomatic. Figure 1 explains the time windows and the fraction infected in the prepatency period especially adjusted to SARS-CoV2. The time windows are specific for a given virus. The index case in most constellations would also be the primary case of a chain of infection. A secondary case would, for instance, be a 1°contact person who becomes positive with or without symptoms. A tertiary case would be a contact person of the 1°contact, e.g., a household member of the 1°contact. Rarely, an index case could be a latter degree case such as a secondary or tertiary case and thus drawing attention to the primary case by backward contact tracing. For pragmatic reasons, we call a 2°contact person becoming positive or symptomatic a tertiary case even when the 1°contact would remain asymptomatic. In analogy to Brockmann and Helbing [8], their wave model on international spread of pathogens via airports can be used to explain the contact chain and classify exposure of 1° and 2°.

To judge the impact of Hh-Q2° within the entire package of isolation and quarantine, the theoretical efficacy ε needed for control and θ, the fraction infected before a case can be identified (including asymptomatic cases), the central formula

0 $${\text{R}}_{{{\text{eff}}}} = \left( {1 - \, \theta } \right)(1 - \varepsilon ){\text{R}}_{0} + \theta {\text{R}}_{0}$$

is used. R_0_ is the basic reproductive number and R_eff_ the effective reproductive number. According to Fraser et al. [1], the ε needed for control (R_eff_ = 1; θ = 0) of a pathogen with a given R_0_ is calculated by the formula

3 $$\varepsilon > 1 - 1/{\text{R}}_{0}$$

(bracket refers to column in Table 2).

The ε_I_ (the ε of isolation) and ε_T_ (the ε of contact tracing and quarantine) together contribute to the effective ε (ε_eff_). Since ε_I_ depends on the manifestation index and the amount of testing carried out, they are not under the control of the CHD. In contrast, ε_T_ in a given county is under the authority of a given CHD and therefore in focus here.

For R_eff_ = 1 and ε = 1, the central formula (0) describes θ with

4 $$\theta {\text{R}}_{0} < \, 1.$$

Since both, ε and θ, impact on ε_eff_ for SARS-CoV2 at the same time, the central formula has to be transformed to calculate ε_eff_. Setting R_eff_ = 1, the control threshold, and replacing R_0_ by 1 – 1/ε_eff_, results in

6 through 10 $$\varepsilon_{{{\text{eff}}}} = \, 1 - \left[ {\left( {1 - \, \theta } \right)(1 - \varepsilon ) \, + \theta } \right].$$

One scenario is calculated for a fraction of asymptomatic {F(asymt)} or missed cases of 20% (manifestation index = 80%; i.e., ε_max_ = 80%) and another of 30% (manifestation index = 70%; i.e., ε_max_ = 70%). The fraction of asymptomatic or missed cases increases with younger age [9]. The fraction of asymptomatic and missed cases in the population obviously dictates the limit of the ε maximal achievable (ε_max_).

$$\varepsilon_{\max } = \, 100\% - {\text{F}}\left( {{\text{asymt}}} \right).$$

The “gap” between the theoretical ε needed for control and the ε maximally achievable given a specific fraction of asymptomatic or missed cases is calculated by gap = ε − ε_max_ (5).

For columns (6) through (10), these calculations have been repeated using gap = ε − ε_eff_.

The gap is a percentage or in case there is no gap, there is a buffer marked as a percentage “+”, i.e., a percentage which theoretically can be afforded not to be ascertained.

The fraction θ, if not intervened by quarantine, obviously is reducing the ε_eff_. Different θ and by quarantine reduced θ (>) are shown in columns (6) through (10). The gap here is calculated as gap = ε − ε_eff_. ε taken from (3). The 95%-confidence intervals are used for continuous variables as well as proportions throughout. For the latter, a normal distribution can be assumed given the size of the numbers.

As the results from Table 2 later demonstrate, reducing θ narrows the gap between ε_eff_ (i.e., ε_I_ + ε_T_) and the theoretical ε needed for control according to ε > 1 – 1/R_0_. To this end, the aim of any control measures must be to reduce θ as much as possible by quarantine orders. Since isolation fails in the asymptomatic or missed cases, it is essential to reduce θ by quarantine of contacts in symptomatic and ascertained cases. Here prepatency period 2 is of practical importance (Fig. 1), i.e., until effective control measures are ordered [5]. In the following, the impact of household quarantine for second degree contacts on reducing θ and thereby preventing the spread of SARS-CoV2 will be assessed using field data from the CHD Ploen.

## Results

Within the study period, encompassing the entire first wave and a part of the second wave, a total of 353 (100%) PCR-confirmed cases were ascertained and all documents could be retrieved including information on the household members: 153 cases could be allotted to the first wave from 9 March to 31 August and 200 cases to the second wave as of 1 September to 8 December, the study end. The mean age in the first wave was 51.6 years [48.52; 54.72], in contrast to 42.1 years [39.19; 44.94] in the second wave.

**Fig. 2:**
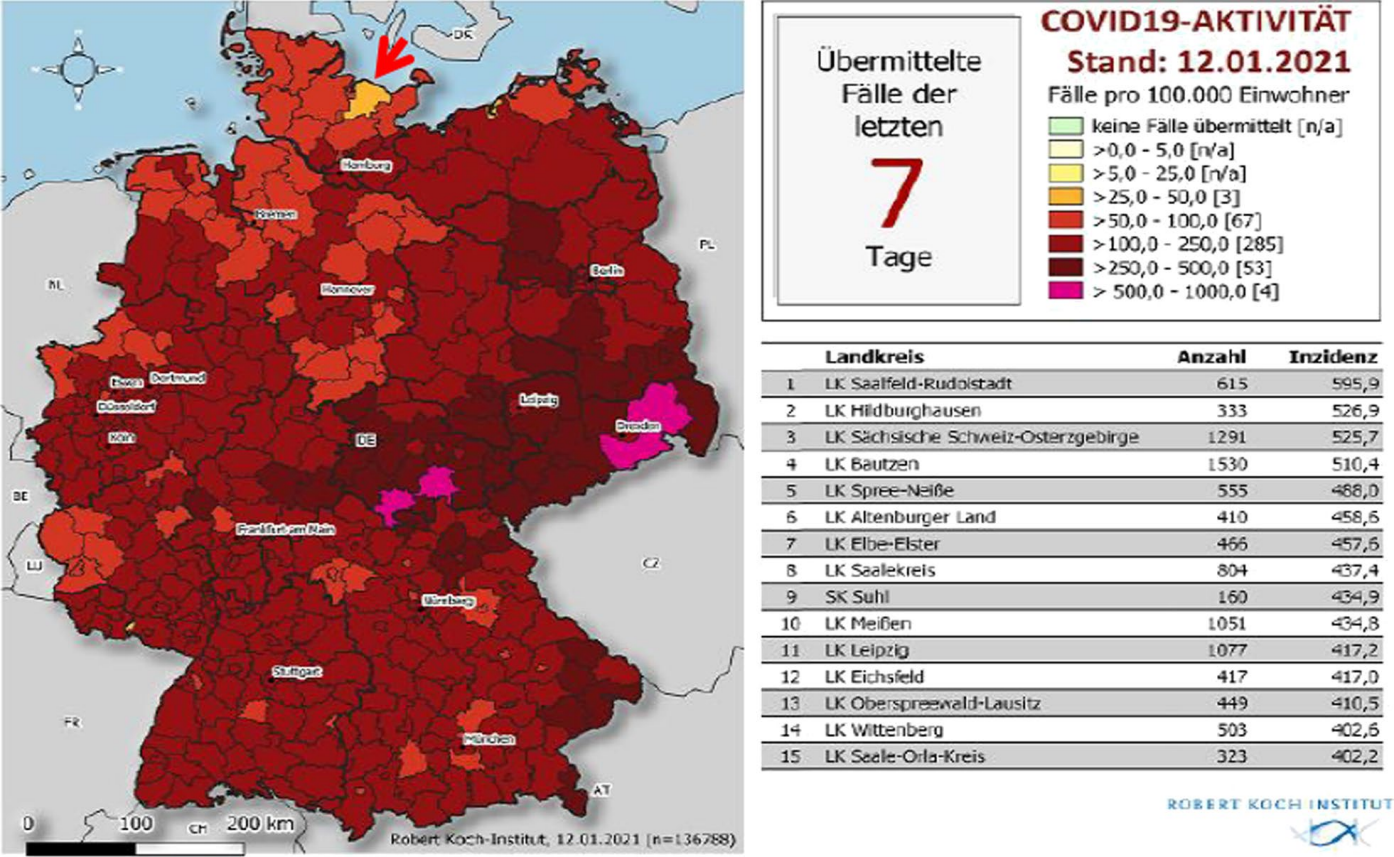
Catchment area and 7-day cumulative incidence within daily report of the RKI on 12 January 2021 [10], arrow marks Ploen county east of the town of Kiel on the Baltic seashore

A total of 225 primary cases were notified to our department based on the German Infectious Diseases Control Act (IfSG) via laboratories carrying out PCR-testing. A variety of test protocols were used in our region and ct-values are not forwarded by all laboratories to the CHDs. Up to the end of this study and beyond the cumulative incidence of SARS-CoV2/COVID-19 cases in Ploen County across the entire pandemic was the lowest in all of Germany (https://interaktiv.tagesspiegel.de/lab/karte-sars-cov-2-in-deutschland-landkreise). Figure 2 illustrates the geographical position of Ploen County and the 7-day cumulative incidence per 100.000 at the time of the peak of the second wave after the lockdown beginning November 2020 [10].

The 225 primary cases came from 219 households and caused the quarantine of 649 individuals, 470 1°contacts and 179 2°contacts. Of the 470 1°contacts, 290 were in case households, 79 were in 63 households that consisted entirely of 1°contacts, and 101 came from 90 households that included 2°contacts. This study focuses on the 179 2°contacts in these 90 households (Table 1). The ratio of primary cases and quarantine orders was 1 to 2.1 (470 by 225) for 1°contacts and 1 to 2.9 (649 by 225) for all quarantine orders including 2°contacts.

**Table 1:** Overview of 353 confirmed cases and type of quarantine ordered from 9 March to 8 December 2020 in the County Health Department Ploen

Cases and contacts by definition	Households exposed	Primary cases	C1° exposed	Secondary cases (C1°positive)	C2° exposed	Tertiary cases (C2° positive)
Primary cases 1°contact (Hh the same as primary case) Hh quarantine based on direct exposure (mean Hh size = (225 + 290)/219 = 2.35)	**219 total**	**225**	**290**	**–**	**n/a**	**n/a**
	56 single	56	**–**	**–**	n/a	n/a
	112 multiple w/o sec. cases	118	188	**–**	n/a	n/a
	51 multiple with sec. cases	51	102	61	n/a	n/a
1°contact (different Hh as primary case) without 2°contacts Hh quarantine based on direct exposure (mean Hh size = (79/63 = 1.25)	**63 total**	**n/a**	**79**		**n/a**	**n/a**
	40 single—no case	n/a	40	**–**	n/a	n/a
	15 single—as case	n/a	15	15	n/a	n/a
	8 multiple—all exposed	n/a	24	10	n/a	n/a
1°contact (different Hh as primary case) with 2°contacts; for the latter Hh quarantine based on indirect exposure (mean Hh size = (101 + 179)/90 = 3.11) (mean Hh size = (8 + 32 + 1 + 5)/9 = 5.11)*	**90 total**	**n/a**	**101**		**179**	
	68 without contact 1° pos	n/a	78	–	111	**–**
	13 with contact 1° pos.only	n/a	14	13	31	-
	8 with contact 2° pos	n/a	8	8	32	20
	1 with contact 2° pos	n/a	1	–**	5	1
Total	372	**225**	470	**107**	179	**21**
**353** cases total (100%)		(63.7%)		(30.3%)		(5.9%)

Hh = household (single vs. multiple, i.e. more than one Hh member); C1° directly exposed contact; C2° indirectly exposed contact

*Household size for the households with converted 2°contacts

**One 1°contact was asymptomatic, not tested but 2°contact in household became symptomatic and confirmed PCR-positive

The indication for Hh-Q2° was triggered by a delay in notification of the CHD by at least 72 h (60–96 h), if the household could not separate itself from the 1°contact or at least partially separate, if younger children or dependents have to be cared for, within this critical time window; or if the 1°contact could not be tested at the time separation within household was considered.

**Fig. 3:**
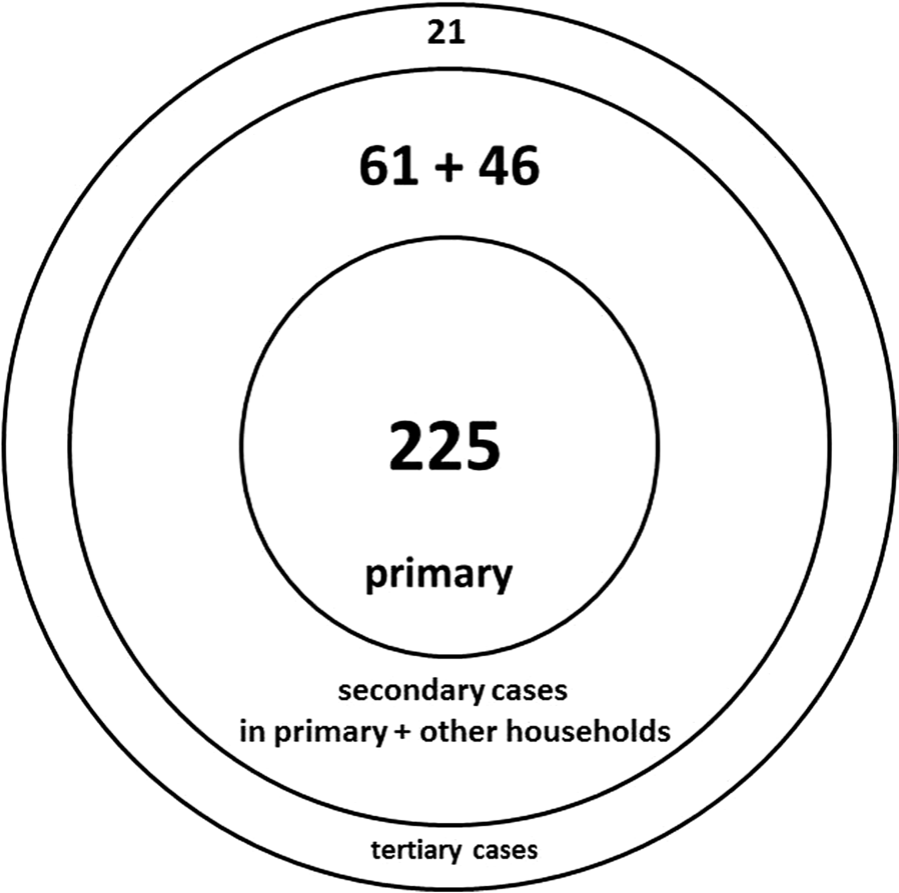
Waves of transmission of 353 confirmed cases form 9 March to 8 December 2020, County Health Department Ploen

Of the 353 cases, there were 225 primary (63.7%), 107 cases (30.3%) among the 1°contacts (secondary cases), and 21 cases (5.9%) among 2°contacts (tertiary cases) (Fig. 3). The risk of infection among the 470 1°contacts was 22.8% [19.01; 26.59] and the risk of infection among the 179 2°contacts was 11.7% [6.99; 16.41]. This means that 1 in 4.4 quarantined 1°contacts and 1 in 8.5 2°contacts evolved into a case. Of the 128 converted contacts, 16.4% occurred among 2°contacts. The efficacy of quarantine in 2°contacts was 51.5% of those in 1°contacts (11.7%/22.8% or 21/179 by 107/470).

The mean household size was considerably larger in the households for which quarantine of 2°contacts was ordered (3.1 members on average). With 5.1 persons per household, the mean was the highest in those households with tertiary cases (Table 1). The 1°contact in one household, consisting of a total of 6 members, remained asymptomatic, while a 2°contact became symptomatic and was PCR-confirmed. This shows that 1°contacts remaining asymptomatic are no guarantee that the virus does not spread further within the household. This is a proof of principle. Immediately after the closure of this study we had another similar constellation in an important institution.

To judge the impact of 5.9% confirmed cases contributed by 2°contacts, a modelling given a fraction asymptomatic or oligosymptomatic (not ascertained) cases of 20% and another of 30% is shown in Table 2. Using Hh-Q2° as an NPI tool with at least 5.9% impact, a situation in a given column such as columns (6) through (9) can be shifted to the right by at least one, possibly even two columns, and thus into a more favourable situation (i.e., better control perspective). Given that 5.9% is the lower margin due to the issue of the manifestation index, the effect is expected to be even greater.

**Table 2:**
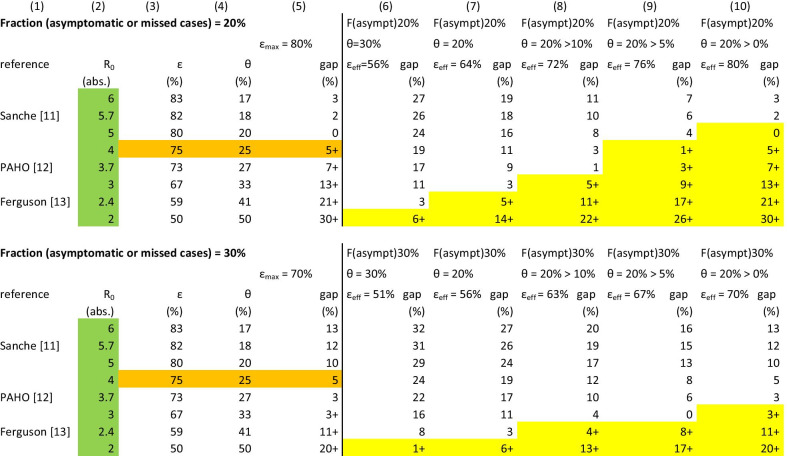
Efficacy (ε) of isolation and quarantine given a fraction (F) asymptomatic (missed cases) and a fraction θ of transmissions before symptom onset

R_0_ = basic reproductive number for SARS-CoV2

ε = efficacy of isolation and quarantine (ε_I_ + ε_T_); (3) ε > 1 – 1/R_0_ according to Fraser et al. [1]

θ = fraction of transmission before symptom onset; (4) θR_0_ < 1 according to Fraser et al. [1]

(5) gap = ε − ε_max_, the difference between ε needed according to ε > 1 – 1/R_0_ (column 3) and ε_max_, given a certain fraction of asymptomatic or missed cases; in case the gap is % + , this means a buffer of allowed insufficiency before ε > 1 – 1/R_0_ is reached

(6) to (10) ε_eff_ resulting out of F(asymptomatic) in the general population + θ the fraction infected before the onset of symptoms; gap = ε − ε_eff_

Percentages are rounded up to the next whole number

## Discussion

The CHD Ploen is one of 15 health departments within the federal state of Schleswig–Holstein (2.8 million). Ploen County (population 128,686) is both a spread-out countryside (59% of the population) and a congested municipal residential area adjacent to the town of Kiel (245,000) with about the other 41% of the population. End of November 2020, Ploen county had a cumulative incidence in the second wave of around 130 per 100.000 and the city of Kiel of 330 per 100.000. There are surely several reasons contributing to this difference which cannot be further addressed here than just to mention that Hh-Q2° is not used in the CHD Kiel.

The first wave started in our region at the beginning of March, 2020. As of 9 March and enhanced at 23 March, lockdown measures were implemented and maintained until the first week of June, 2020. Already on 17 March, the epidemic curve had started to flatten. During the summer, more and more restrictions were lifted and even big events were allowed. In August and September, travel- associated issues were on the forefront until the first week of October, when the second wave set in. As of 2 November, a ‘lockdown light’ was implemented until 16 December when another strict lockdown was ordered.

Exposure and exposure measurement by contact tracing as a form of personal interview are key in field epidemiology [14]. How many persons to be put into quarantine depends obviously upon several factors such as contact pattern, intensity of contact, time axis, strategy towards direct and indirect contacts, societal structure, i.e., fraction of single household and household size, and particularly on the precision of the work of a CHD. The indication for Hh-Q2° was triggered by a delay of notification of the CHD by at least 72 h (60–96 h). This is twice the range of the minimum latency period of SARS-CoV2 (Fig. 1) [2, 7, 12]. Obviously this time window would need more modelling based on a larger sample size, but according to our practical experience, it seems to work. PCR testing at decision points in contact tracing and for separation within households is of great value.

The fraction of single households in our cohort was 29.8% (111 out of 372) and mirrors the societal structure with fewer families and children in general. With 2.1 (1°contacts only) and 2.9 (1° and 2°contacts) persons per primary case put into quarantine, this should be an acceptable burden for society and is much less than that modelled by Aleta et al. [15] and Hinch et al. [16] or in the other CHDs in our region.

The efficacy of Hh-Q2° with 51.5% of the efficacy of quarantine in 1°contact was surprisingly high and is obviously influenced by the quality of the investigations by the CHD and the number of Hh-Q2° ordered (the denominator). Household size appears to be a major risk factor for conversion of contacts into cases or ascertainment of converted contacts as described also by a seroprevalence study in Sweden [17]. Adolescents and young adults were playing an increasing role in the second wave as 1°contact to households with further members according to the “heat chart” of age-specific attack rates over time [10]. Persons in this age group to a large extent still live in the parental home with the original family.

In this study, 16.4% of all converted and symptomatic cases in quarantined persons were ascertained via Hh-Q2°. This means that 1 in 6 converted cases were additionally prevented from spreading the infection further within the community. The all-over impact of Hh-Q2° detecting and containing 5.9% of all cases (21 out of 353) seems large enough to justify the effort in ordering quarantine for 2°contacts.

Hh-Q2° to prevent tertiary cases used early in an outbreak or a pandemic wave can make an impact and increase the efficiency of NPI. The early seeding of chains of infection can be prevented by Hh-Q and makes the virus to run into a dead-end. Hh-Q2° on a comprehensive scale is the preferable option in contrast to a lockdown of the general population. With Hh-Q2°, a lockdown might be prevented or at least significantly delayed as also assumed by Aleta et al. [15]. To further justify Hh-Q2°, investigations within backward contact tracing must be as accurate and as rapid as possible to tailor the quarantine orders, including Hh-Q2°, only to the fraction of the contact pattern in which exposure is most likely. Again this depends upon the quality and efficiency of the work of the CHD. In spite of using Hh-Q2° as a tool in the CHD Ploen, the ratio of cases to quarantine orders was lower than in other CHDs in the region. The ultimate goal is to raise ɛ in spite of the counterproductive viral characteristics (Fig. 1). The manifestation index is subject to the virus-host interaction; the ascertainment of cases in the population in general depends upon the degree of testing; but the management of quarantine and the use of Hh-Q2° are under the authority of the CHD.

In analogy to Brockmann and Helbing [8], the spread at the local level and even in the household setting can be regarded in the same way (Fig. 3). The close contact would be the first wave, the hub, knocking on the door of the non-case household. If the household is not stratified in time, the incubation of the entire household or setting continues in case the contact 1° starts to shed and evolves into a case. The latter can only partially be identified in time, given the key parameters of SARS-CoV2 such as θ including the fraction asymptomatic but infectious subjects.

In general, the household as an entity and endpoint of public health considerations has so far only been partially recognized and accepted, since our health care thinking is to far extent focused on individual aspects. John Oxford [Vienna conference “Influenza Vaccines for the World”, 18 to 20 October 2006] pointed out for the first time and on many occasions thereafter that the 1918 pandemic (“Spanish Flu”) was primarily a tragedy of families. Once the virus entered a family, the death toll was significant. The virus enters into families via one family member (a 1°contact) having had an efficacious contact outside. In analogy to the Japanese cluster approach for backward contact tracing [18], Hh-Q2° could be seen as the equivalent forward-orientated control approach.

All measures taken within the bundle of NPI also have to be seen in the context of compatibility with social aspects and thus raising acceptability and compliance. Ordering Hh-Q, at least due to the current regulation in Germany, is synergistic, since parents with children under quarantine, for example, do not have to bother about sick-leave or any other option to justify staying at home, since a quarantine order entitles them to social security and compensatory salary payment. The main argument, however, remains the public health intervention and blocking the chain of transmission. The conversion of the 1°contact into a case with shedding at least 2 days before symptom onset or as an asymptomatic spreader is the cornerstone of the argument for Hh-Q2°. According to our observations, about one in 10 tertiary cases occurs without symptoms in the close contact of that household.

The inability and time delay of detection of this conversion with onset of viral shedding caused by the fraction θ including the fraction of asymptomatic, are facts and surveillance of 1°contact by health departments is necessarily inefficient due to the time and shedding characteristics of SARS-CoV2. The most recent data of Zhang et al. [19] makes this effort appear even more inefficient since they pointed out that even with tight testing of household members in quarantine, many are missed as their serological data revealed. Furthermore, the numbers in quarantine increase rapidly in a pandemic wave and the manpower bound by active surveillance accumulates accordingly. This manpower could better be used otherwise, for instance in ambulatory testing of clusters.

Since incubation of the household continues over the entire period of a 1°contact, if it starts to shed virus, the current 14 days of quarantine are supportive for the efficacy of Hh-Q2°. A shortening of the quarantine duration from 14 to 10 days could have a detrimental impact, since the 14 days so far, guaranteed to most extend that 2°contacts would still be in quarantine at the time of being transmissible, whether symptomatic or not, after being infected by a 1°contact.

Finally, all this has to be driven by the motivation to contain or flatten the pandemic wave to protect the vulnerable but still limiting the burden for the general society as much as possible. The tool of Hh-Q2° is easy to order and logical at the same time. It is astonishing that it was widely overlooked and not identified, at least by authorities, as a straightforward measure within the tool box of NPI. So far it has only been addressed by modellers [15, 16]. Across the entire sessions concerning SARS-CoV2 and COVID-19 during the ESCAIDE conference on 26 and 27 November 2020 organised by the ECDC, it was only mentioned on one slide within the keynote lecture by George Gao, head of the Centre for Disease Control China, in regard to lessons learned in China [20]. As demonstrated within the study presented here, Hh-Q2° is also feasible in Western countries. Interestingly the concept of ring vaccination in the endgame of the smallpox eradication campaign was based on the same principle—in this case vaccination of the household members of 1°contact persons of a case (Adam Finn, 39th Annual Conference of the European Paediatric Infectious Diseases Society). We advocate giving Hh-Q2° a higher priority within the tool box of NPI, at least for the control of SARS-CoV2, as already reported by Aleta et al. [15]. Whether it is more widely used than made public, remains an open issue. If explained to persons to be put under Hh-Q, it is widely accepted and plausible. Hh-Q2° is to a greater extent not yet addressed in national guidelines since it is a field approach and is easily overlooked by national authorities. The RKI should urgently integrate the approach demonstrated here into their national guidelines. The tool of Hh-Q2° is both logical and straightforward.

### Limitations

The study design was retrospective, but the documentation of the source population was sufficiently detailed. The power of the study was limited given the size of the local population and the low incidence. Household members were either tested negative or not tested supposedly due to lack of symptoms. This mirrors a real-life situation. One could speculate that Hh-Q2° was even much more efficacious since it also prevented asymptomatic persons within the 2°contacts from transmitting beyond their own household. This would mean that the impact of household quarantine was underestimated in this study by at least 20%. The eligibility for Hh-Q2° might have been selective, but is a standard option in our department. Finally, the base for the case definition could be limited by false-positive PCR tests. The strength of the study presented here, however, is that these are real-life data and practical issues around them are addressed instead of modelling with varying assumptions.

## Conclusions

Given the impact of Hh-Q2° and the output of tertiary cases from the number of persons put into quarantine, Hh-Q2° is an effective tool to increase the efficacy of quarantine measures. It should be used more readily after detailed investigations of the contact pattern and timelines to overtake the virus in its spread. Hh-Q2° can even be critical for prevention or containment of local outbreaks of SARS-CoV2. We believe we have identified a common gap within the portfolio of NPI measures which can be easily implemented and carried out in a differentiated or crude form. It is unlikely that the cumulative incidence in Ploen County is the lowest in Germany just by chance.

The pandemic revealed weaknesses in the local health departments which have to be dealt with as fast as possible. In the meantime, the available manpower must be used as efficiently as possible. Inefficient approaches have to be omitted immediately. Hh-Q2° is as simple and straightforward as we wish other interventions to be. The burden for a few should be acceptable in favour of avoiding or at least postponing measures for the entire society such as lockdowns.

Addendum: Due to the lengthy review process time has gone bye and the role of NPI has changed due to high vaccine coverage rates in many countries. Hh-Q2° should be recognized as effective tool for future pandemics.

## Data Availability

The datasets generated and analysed during the current study are not publicly available but are available from the corresponding author on reasonable request after de-identification.

## Abbreviations

CHD: County health department
Hh-Q2°: Household quarantine in 2°contacts in household different form the case household
NPI: Non-pharmaceutical interventions
1°contact: A close (direct) contact
2°contact: A (indirect) contact to a close contact
